# Spousal associations between social participation and chronic diseases among Chinese middle-aged and older adults: an actor-partner interdependence model analysis

**DOI:** 10.3389/fpubh.2025.1576933

**Published:** 2025-08-08

**Authors:** Yunchen Ruan, Yan Li, Qingbao Wang

**Affiliations:** School of Humanities and Social Sciences, Fuzhou University, Fuzhou, Fujian Province, China

**Keywords:** social participation, spouse, chronic diseases, actor-partner interdependence model, middle-aged and older adults, China

## Abstract

**Objectives:**

While previous studies have explored the link between a spouse’s social participation and their partner’s general physical health among Chinese middle-aged and older adults, none have examined its association with partner chronic disease or potential gender differences. Our study aims to address this gap.

**Methods:**

This study used data from the 2013, 2015, and 2018 waves of the China Health and Retirement Longitudinal Study (CHARLS). The sample comprised 3,072 couples where both partners were aged 45 or older. Chronic disease was defined as the count of self-reported chronic conditions. Social participation was measured as the number of distinct social activity types engaged in. Analyses employed the actor-partner interdependence model (APIM).

**Results:**

Spousal social participation was significantly associated with partner chronic disease, with significant gender differences. Specifically, the wife’s social participation was positively associated with the husband’s chronic diseases, whereas the husband’s social participation was negatively associated with the wife’s chronic diseases. Furthermore, the wife’s depressive symptoms significantly mediated both of these associations.

**Conclusion:**

Spousal social participation is associated with partner chronic disease among Chinese middle-aged and older adults, with the nature of this association varying significantly by gender. These findings suggest that developing and strengthening social support networks, alongside implementing gender-sensitive interventions to promote social participation, could improve health outcomes.

## 1 Introduction

Social participation is central to the concept of active aging, addressing the growing challenges posed by population aging. Research has consistently identified it as a key protective factor for individual health (1–3). For example, it can enhance chronic disease management by strengthening social connections and boosting self-efficacy (4). Studies have emphasized the interdependence in interpersonal relationships (5–8), particularly between caregivers and care recipients (9). This interdependence also extends to spouses, where one spouse’s social participation can directly or indirectly influence the health of the other (10), especially in terms of emotional and psychological well-being (11, 12). Thus, exploring the relationship between social participation and health necessitates moving beyond an individual perspective and focusing on spousal associations, specifically the partner effect.

Research has indicated that social participation is associated with individual health, quality of life, and functional ability (13). Notably, the positive impact of social participation on health is most pronounced among middle-aged and older adults (14, 15). Although some studies have explored the positive effects of social participation on individual health (16–19), most have concentrated on the individual level, while neglecting the partner effect between spouses (20, 21). Current literature examining health issues among middle-aged and older adults using the Actor-Partner Interdependence Model (APIM) remains limited (22). A study utilizing CHARLS 2018 and 2020 data investigated longitudinal dyadic associations between internet use and mental health, revealing that although both husbands’ and wives’ internet use demonstrated significant actor effects on their depressive symptoms, the partner effect was observed exclusively among wives (23). Furthermore, more in-depth analysis is needed to explore gender differences in the impact of social participation on chronic diseases in partners. Existing research indicates significant gender differences in the effects of social participation on physical health (24). Women are more likely than men to seek emotional support through social participation within social networks (25). In contrast, men tend to enhance their psychological resilience through role identity (26, 27).

This study utilized data from the China Health and Retirement Longitudinal Study (CHARLS) to explore the association between spousal social participation and chronic diseases, addressing the aforementioned knowledge gaps. We established two partner effect pathways to examine gender differences: the association of the wife’s social participation with the husband’s chronic diseases and the association of the husband’s social participation with the wife’s chronic diseases. Additionally, we analyzed the mediating mechanisms underlying these observed partner associations. Spouses’ social participation is associated with chronic diseases in their partners among Chinese middle-aged and older adults, with this association being gender-dependent.

## 2 Materials and methods

### 2.1 Study participants

The CHARLS is a nationwide household survey organized by the National School of Development at Peking University, covering 28 provinces (including municipalities and autonomous regions), 150 counties, and 450 villages. The survey targeted Chinese adults aged 45 years and older, along with their spouses. Ethical approval was obtained from the Peking University Ethical Review Committee (IRB0000105211015), and all participants provided written informed consent.

**Figure 1:**
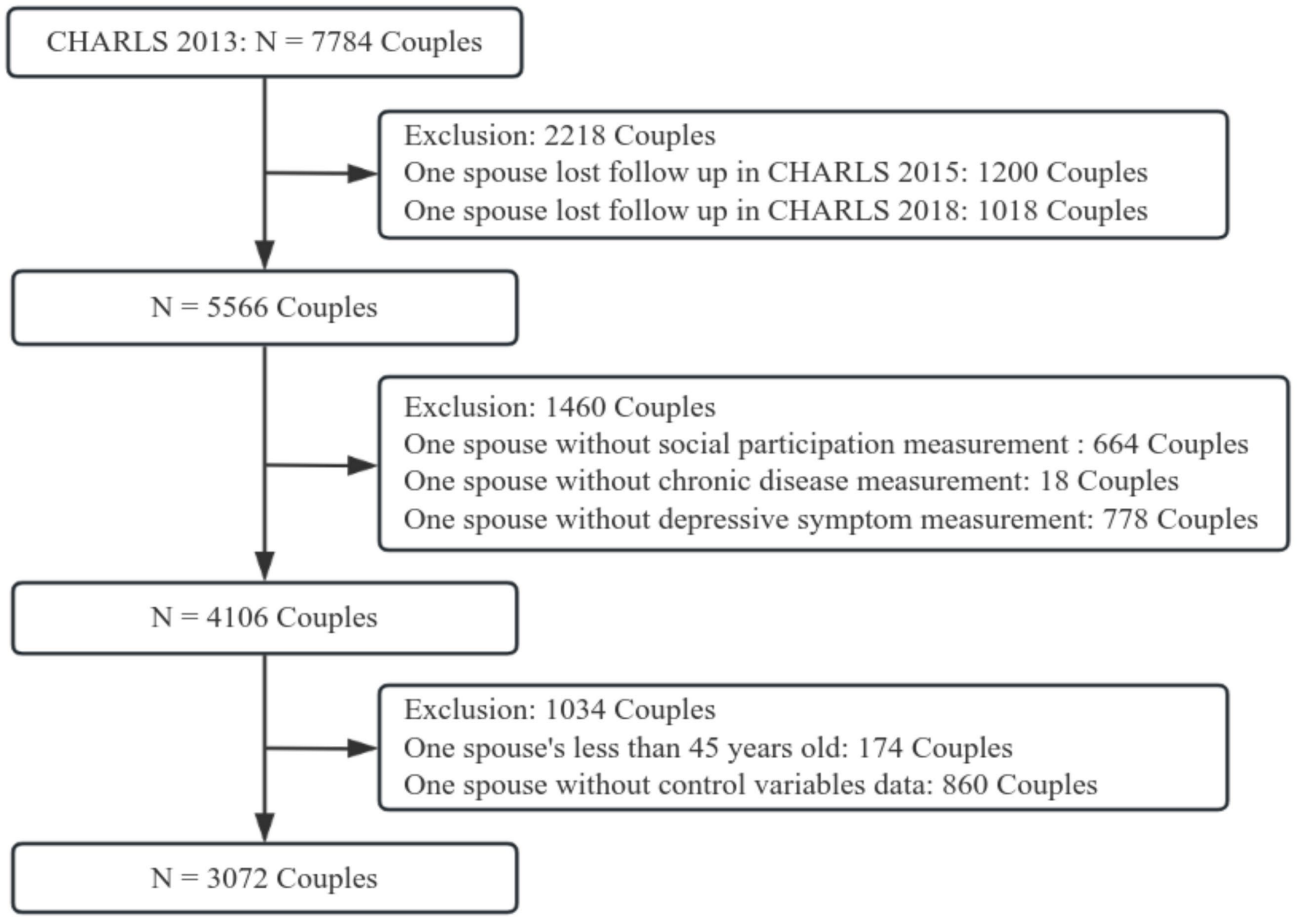
Flow chart of analytic sample.

Our study was based on CHARLS data from 2013, 2015, and 2018. As shown in Figure 1, a total of 5,566 couples participated in the three rounds of the survey. After excluding couples with missing values for social participation (*N* = 664), chronic diseases (*N* = 18), depressive symptoms (*N* = 778), and covariates (*N* = 860), as well as individuals under the age of 45 (*N* = 174), a final sample of 3,072 couples was used for analysis.

### 2.2 Chronic diseases

According to prior research (28), chronic diseases were assessed through 14 self-reported conditions using the question: “Have you been diagnosed by a doctor with any of the following diseases: hypertension, dyslipidemia, diabetes or high blood sugar, cancer or malignant tumor, chronic lung disease, liver disease, heart disease, stroke, kidney disease, stomach or other digestive system diseases, arthritis or rheumatism, emotional or mental disorders, memory-related diseases, or asthma?” The total number of chronic diseases for each respondent was then calculated. The chronic disease score ranged from 0 to 14, with higher scores indicating a greater number of chronic diseases.

### 2.3 Social participation

Social participation was determined based on respondents’ self-reported engagement in social activities over the past month. These activities included whether they had: (1) interacted with friends; (2) played mahjong, chess, or cards, or visited community clubs; (3) provided help to family members, friends, or neighbors who do not live with them and have not paid for assistance; (4) participated in sports, social, or other types of clubs; (5) joined community-related organizations; (6) engaged in volunteer or charity work; (7) cared for sick or disabled adults who do not live with them and have not paid for caregiving; (8) attended educational or training courses; (9) engaged in stock investments; (10) used the internet; or (11) participated in other activities. The total number of social activities was calculated using social participation scores, ranging from 0 to 11, with higher scores reflecting increased involvement in a diverse range of social activities.

### 2.4 Depressive symptoms

Depressive symptoms were measured using the Center for Epidemiologic Studies Depression Scale – 10 items (CES-D-10), which includes 10 questions assessing the respondents’ feelings and behaviors over the past week. Eight questions focus on the frequency of experiencing various depressive symptoms, while the remaining two assess the frequency of positive emotions during the same period. Each response option is assigned a value, and the scores for all 10 questions are summed (with two items reverse-scored). The scoring system is based on the number of days the participant experienced negative and positive emotions in the past week. For example, ‘Rarely or none of the time (less than 1 day)’ is assigned 0 points, ‘Some or a little of the time (1–2 days)’ is assigned 1 point, ‘Occasionally or a moderate amount of the time (3–4 days)’ is assigned 2 points, and ‘Most or all of the time (5–7 days)’ is assigned 3 points. The total score ranges from 0 to 30, with higher scores indicating greater levels of depressive symptoms.

### 2.5 Covariates

The covariates included the respondents’ age, Activities of Daily Living (ADL) status (0 = disability, 1 = no disability), work status (0 = employed, 1 = unemployed), and household socioeconomic status. Household socioeconomic status was measured by region (1 = rural, 0 = urban) and household assets, calculated as the total number of 17 luxury items owned by the household (e.g., television, computer, mobile phone).

### 2.6 Statistical analysis

**Figure 2:**
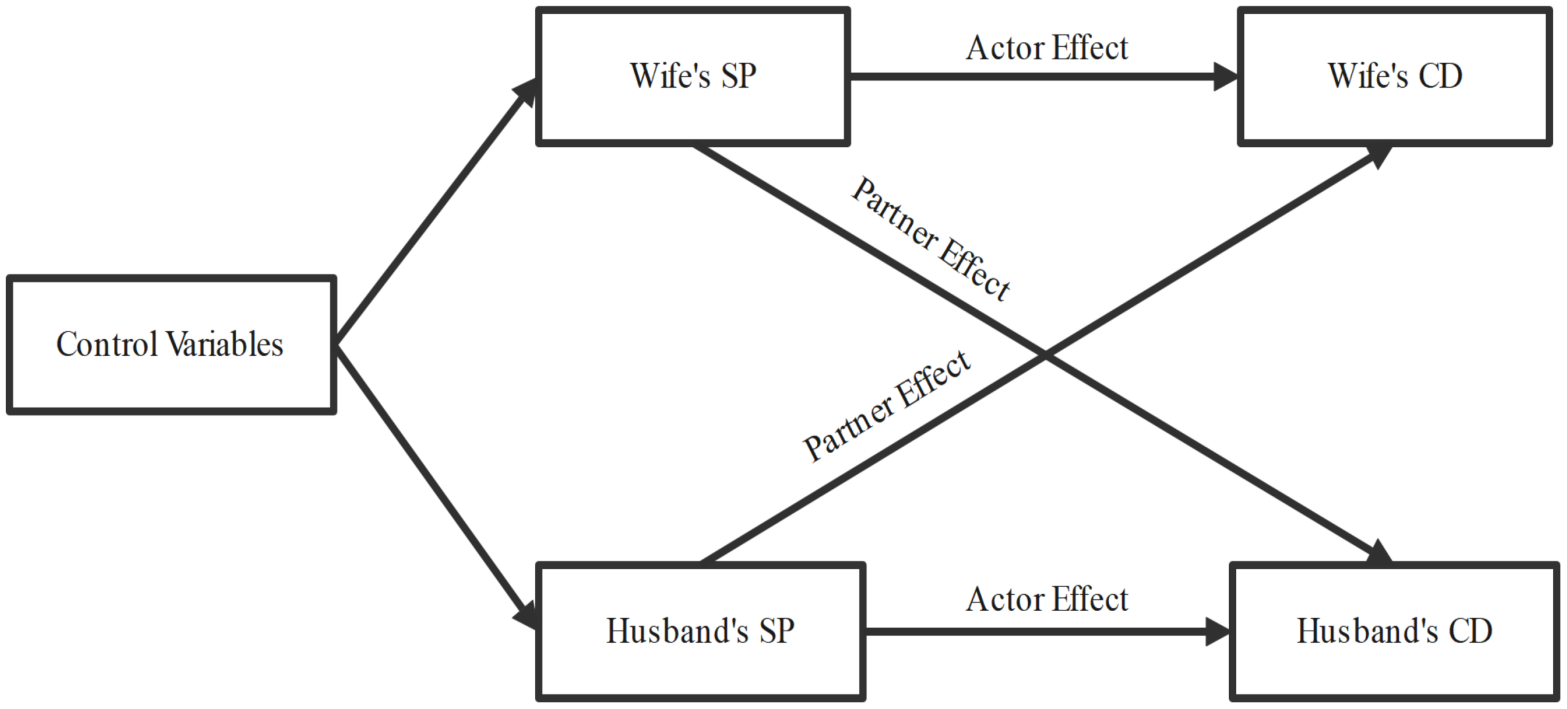
Framework of actor-partner interdependence model. SP, social participation; CD, chronic disease.

First, the study established the analytical foundation using Ordinary Least Squares (OLS) regression to explore the association between spousal social participation and chronic diseases. Model 1 examined the relationship between wives’ social participation and husbands’ subsequent chronic diseases, while Model 2 analyzed the relationship between husbands’ social participation and wives’ subsequent chronic diseases. To investigate the relationship between spouses’ social participation and the number of chronic diseases, we employed the Actor-Partner Interdependence Model (APIM) (29). APIM is a well-established methodology for analyzing dyadic data, designed to examine how an individual’s characteristics influence their own outcomes (actor effects) and their partner’s outcomes (partner effects). Structural equation modeling is recommended as a conceptually straightforward methodology for testing APIM (30). As shown in Figure 2, we estimated four paths in the model: two actor effect paths (the association of the wife’s and husband’s social participation with their own subsequent chronic diseases) and two partner effect paths (the association of the wife’s and husband’s social participation with their partner’s subsequent chronic diseases). Finally, longitudinal mediation models were used to examine the mediating role of depressive symptoms in the association between spousal social participation and chronic diseases, exploring potential mechanistic pathways within these observed relationships. Two longitudinal mediation models were employed: Model 1 included the wife’s depressive symptoms as the mediator, while Model 2 included the husband’s depressive symptoms as the mediator. APIM and bootstrapped mediation analyses were conducted in R using the “lavaan” packages.

## 3 Results

### 3.1 Basic characteristics of the study population

Participant characteristics are presented in Table 1. Husbands reported slightly higher levels of social participation than wives. In contrast, chronic diseases and depressive symptoms were more prevalent among wives than husbands. These differences highlight potential gender disparities in health outcomes and social engagement. Supplementary Table S1 shows longitudinal trends for key variables (2013–2018): for chronic diseases, wives’ mean increased from 1.36 (2013) to 1.54 (2015) and 2.43 (2018), while husbands’ mean increased from 1.25 (2013) to 1.40 (2015) and 2.25 (2018).

**Table 1:** Descriptive statistics of study participants at baseline.

Variables	Wife (*n* = 3,072); N (%) or Mean (SD)	Husband (*n* = 3,072); N (%) or Mean (SD)
Social participation	0.91 (1.06)	1.04 (1.15)
Chronic disease	1.36 (1.37)	1.25 (1.32)
Depressive symptoms (2015)	8.78 (6.57)	6.57 (5.76)
Age	57.08 (7.55)	59.04 (8.06)
ADL		
Disability	26.01%	17.84%
Non-disability	73.99%	82.16%
Working status		
Employed	71.52%	81.41%
Unemployed	28.48%	18.59%
Region		
Rural	82.03%	76.89%
Urban	17.97%	23.11%
Household assets	5.22 (2.23)	

Continuous and categorical variables were expressed as mean (standard deviation) and number (proportion), respectively.

### 3.2 Relationship between spousal social participation and chronic diseases

Table 2 presents the OLS regression results examining the association between spousal social participation in 2013 and subsequent chronic diseases in middle-aged and older adults in 2018. The statistical results revealed a significant association between spousal social participation and chronic diseases in middle-aged and older adults, with notable gender differences. Model 1 demonstrated a significant positive association between the wife’s social participation and the husband’s subsequent chronic diseases (*p* < 0.01), where higher levels of wife’s social participation corresponded to a higher number of chronic diseases in the husband (*β* = 0.092, *p* < 0.01). Model 2 revealed a significant negative association between the husband’s social participation and the wife’s subsequent chronic diseases (*p* < 0.05), where higher levels of husband’s social participation corresponded to fewer chronic diseases in the wife (*β* = −0.067, *p* < 0.05).

**Table 2:** Associations between spouse’s social participation and partner’s chronic disease.

Variables (Reference)	Husband’s chronic disease; model 1			Wife’s chronic disease; model 2		
	Coefficient	*p* value	SE	Coefficient	*p* value	SE
Wife’s social participation	0.092**	0.004	(0.032)	-	-	-
Husband’s social participation	-	-	-	−0.067*	0.026	(0.030)
Covariates						
Age	0.017***	0.000	(0.005)	0.025***	0.000	(0.005)
ADL (Disability)	−0.884***	0.000	(0.088)	−1.055***	0.000	(0.076)
Working status (Employed)	0.518***	0.000	(0.095)	0.468***	0.000	(0.080)
Region (Rural)	0.156	0.070	(0.086)	0.279**	0.003	(0.093)
Household assets	−0.038*	0.019	(0.016)	−0.031	0.058	(0.016)
Intercept	1.974***	0.000	(0.318)	1.811***	0.000	(0.311)
*R* ^2^	0.077			0.108		
Sample size	3,072			3,072		

*p < 0.05, **p < 0.01, ***p < 0.001.

### 3.3 Longitudinal analysis of spousal social participation on chronic diseases

**Figure 3:**
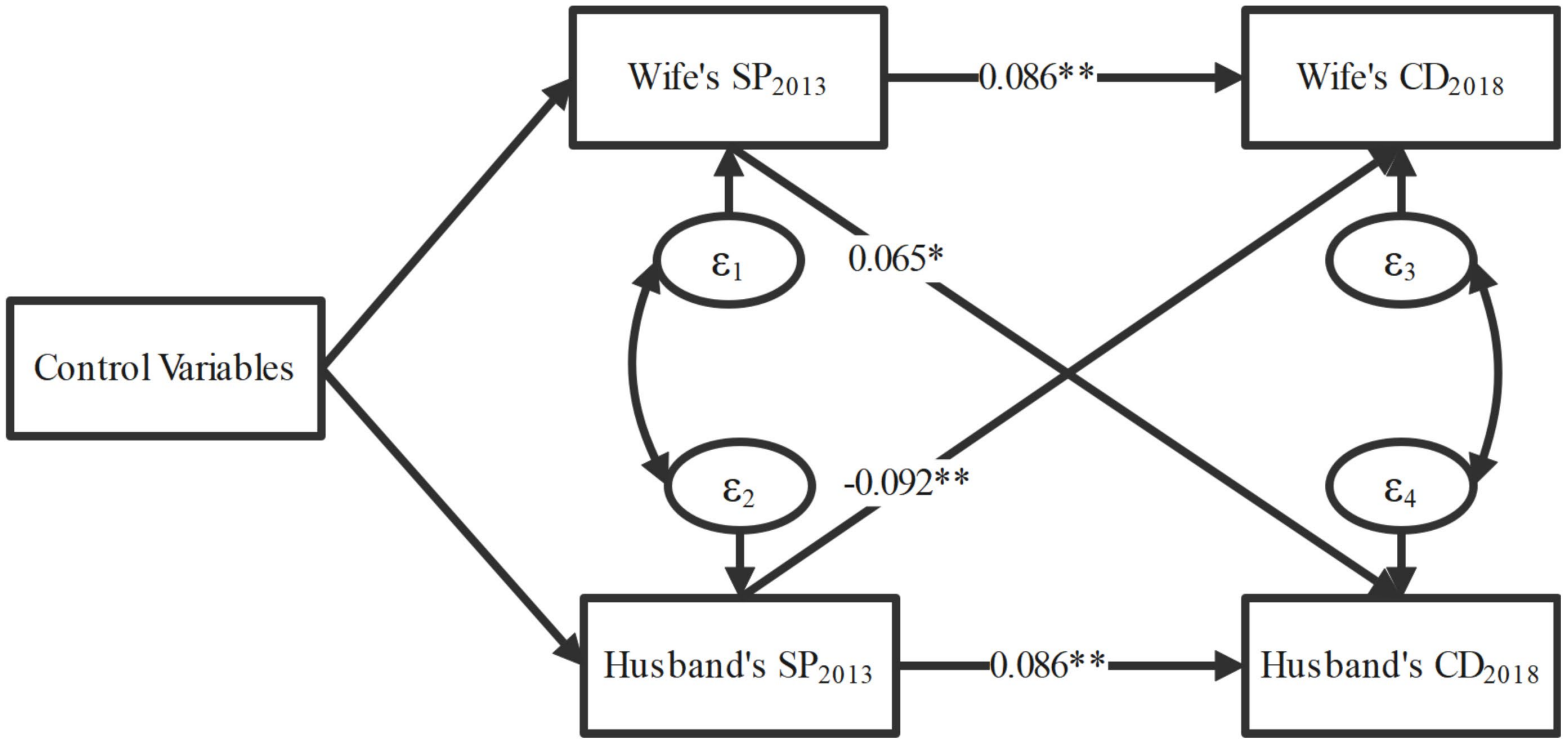
Results of the actor-partner interdependence model. The model fit indices of the figure and the coefficients of all paths are presented in Supplementary Table S2; adjusted for age, region, ADL, working status, and household assets; SP, social participation; CD, chronic disease; ADL, activities of daily living; *p < 0.05, **p < 0.01, ***p < 0.001.

Figure 3 illustrates the results of the Actor-Partner Interdependence Model (APIM), analyzing the relationship between social participation levels in 2013 and subsequent chronic diseases in 2018. The findings confirmed both actor and partner effects of social participation with chronic diseases. The paths linking a wife’s or husband’s social participation to their spouse’s chronic diseases represent partner effects. The model fit indices of Figure 3 and the coefficients of all paths are presented in Supplementary Table S2. The APIM revealed a significant association between spousal social participation and chronic diseases in middle-aged and older adults, with a gender difference. Higher levels of the wife’s social participation were associated with a subsequent increase in the number of chronic diseases in her husband (*β* = 0.065, *p* = 0.048). Conversely, higher levels of the husband’s social participation were associated with a subsequent decrease in the number of chronic diseases in his wife (*β* = −0.092, *p* = 0.003). Actor effects represent the paths linking a wife’s or husband’s social participation to their own chronic diseases. In other words, these associations reflect the relationship between an individual’s social engagement and their personal health outcomes. Social participation showed significant associations with individuals’ subsequent chronic diseases (wife: *β* = 0.086, *p* = 0.009; husband: *β* = 0.086, *p* = 0.005).

### 3.4 Longitudinal mediation analysis of spousal social participation on chronic diseases

**Figure 4:**
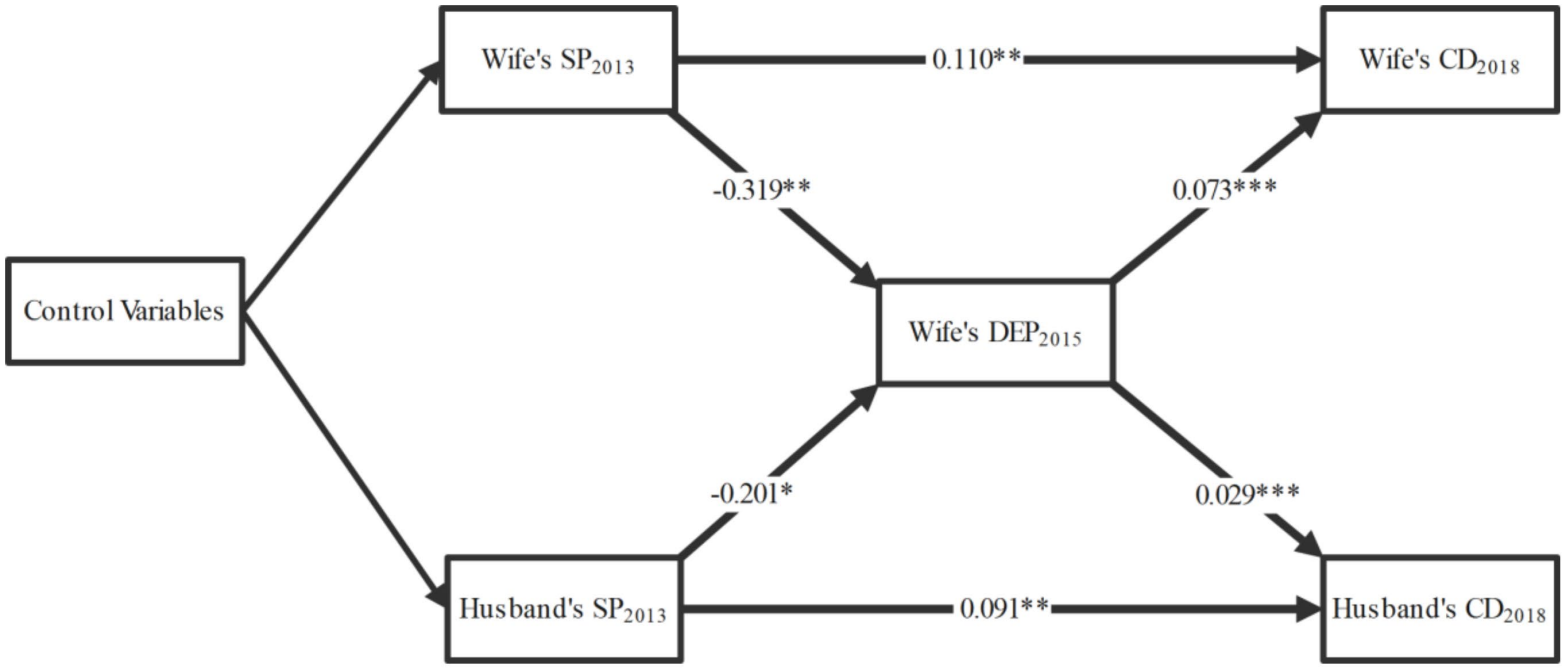
Mediating effects of wife’s depressive symptoms. The model fit indices of the figure and the coefficients of all paths are presented in Supplementary Table S3; adjusted for age, region, ADL, working status, and household assets; SP, social participation; CD, chronic disease; DEP, depressive symptoms; ADL, activities of daily living; *p < 0.05, **p < 0.01, ***p < 0.001.

**Figure 5:**
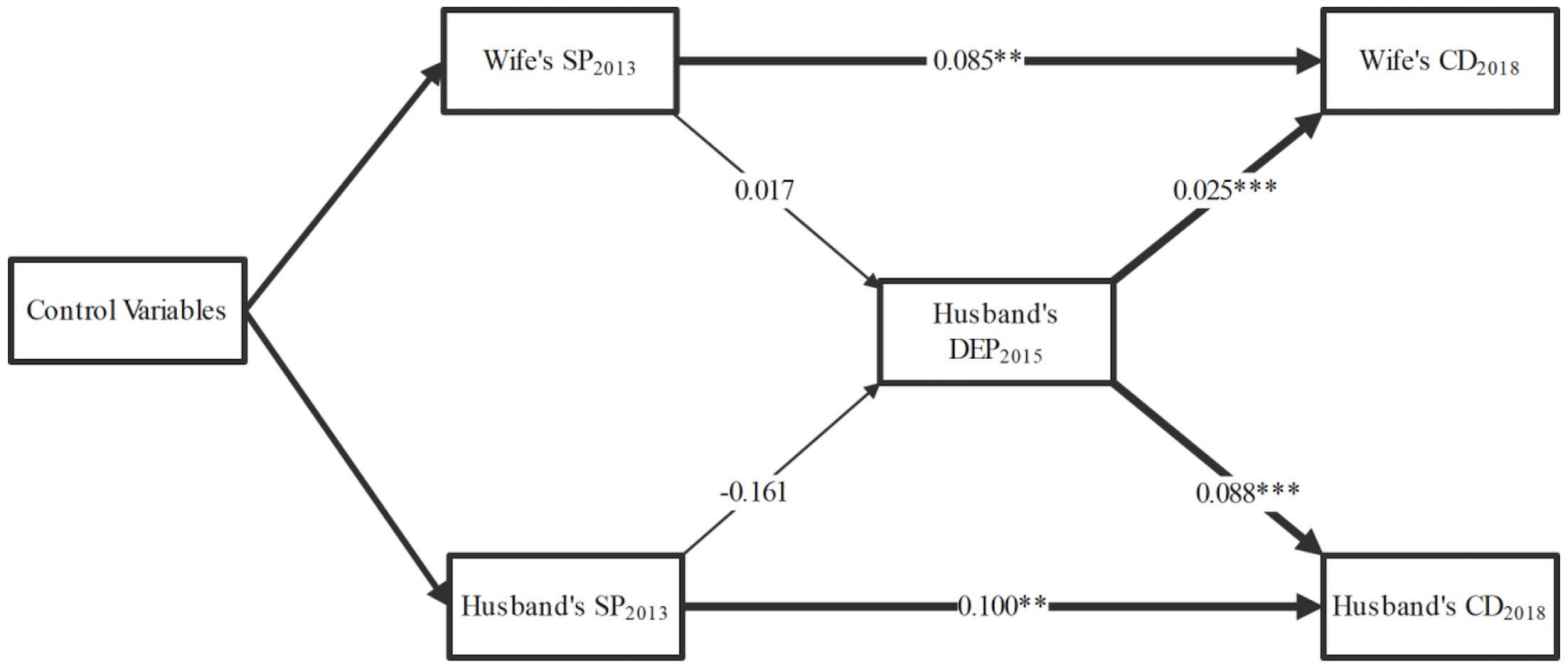
Mediating effects of husband’s depressive symptoms. The model fit indices of the figure and the coefficients of all paths are presented in Supplementary Table S4; adjusted for age, region, ADL, working status, and household assets; SP, social participation; CD, chronic disease; DEP, depressive symptoms; ADL, activities of daily living; *p < 0.05, **p < 0.01, ***p < 0.001.

Figure 4 presents the longitudinal mediation model with the wife’s depressive symptoms in 2015 as a mediator in the relationship between social participation and chronic diseases among spouses. The model fit indices of Figure 4 and the coefficients of all paths are presented in Supplementary Table S3. The wife’s social participation in 2013 was significantly associated with her depressive symptoms in 2015 (*β* = −0.319, *p* = 0.004), which, in turn, was associated with the husband’s chronic diseases in 2018 (*β* = 0.029, *p* = 0.000). Similarly, the husband’s social participation in 2013 was significantly associated with the wife’s depressive symptoms in 2015 (*β* = −0.201, *p* = 0.046), which subsequently was associated with her chronic diseases in 2018 (*β* = 0.073, *p* = 0.000). To assess the mediation effects, the Bootstrap method was used to calculate statistical significance (Table 3). The wife’s depressive symptoms in 2015 played a significant mediating role in the association between the wife’s social participation in 2013 and the husband’s chronic diseases in 2018 (*β* = −0.009, *p* = 0.012, 95% CI = [−0.017, −0.003]). Additionally, in the association between the husband’s social participation in 2013 and the wife’s chronic diseases in 2018, the mediating role of the wife’s depressive symptoms in 2015 was significant (*β* = −0.015, *p* = 0.049, 95% CI = [−0.031, −0.001]). Furthermore, in Figure 5, we also examined the mediating role of the husband’s depressive symptoms in 2015, but found it not significant (*β* = 0.017, *p* = 0.861; *β* = −0.161, *p* = 0.074). Specific details of Figure 5 and the Bootstrap mediation analysis of husbands’ depressive symptoms are provided in Supplementary Table S4 and Table 4, respectively.

**Table 3:** Bootstrap results of the mediating effects analysis of wife’s depressive symptoms.

Effect estimate	*β*	95% Bootstrap CI	*p* value
Indirect Effect 1	−0.009	−0.017 to −0.003	0.012
Indirect Effect 2	−0.015	−0.031 to −0.001	0.049
Direct Effect 1	0.075	0.013 to 0.152	0.037
Direct Effect 2	−0.077	−0.134 to −0.018	0.009
Direct Effect 3	0.110	0.048 to 0.170	0.001
Direct Effect 4	0.091	0.028 to 0.152	0.004
Total Indirect Effect	−0.024	−0.040 to −0.010	0.001
Total Direct Effect	0.199	0.093 to 0.327	0.001
Total Effect	0.175	0.070 to 0.302	0.002

Indirect Effect 1: Wife’s SP → Wife’s DEP → Husband’s CD; Indirect Effect 2: Husband’s SP → Wife’s DEP → Wife’s CD; Direct Effect 1: Wife’s SP → Husband’s CD; Direct Effect 2: Husband’s SP → Wife’s CD; Direct Effect 3: Wife’s SP → Wife’s CD; Direct Effect 4: Husband’s SP → Husband’s CD. SP, social participation; CD, chronic disease; DEP, depressive symptoms.

**Table 4:** Bootstrap results of the mediating effects analysis of husband’s depressive symptoms.

Effect estimate	*β*	95% Bootstrap CI	*p* value
Indirect Effect 1	0.002	−0.015 to 0.020	0.862
Indirect Effect 2	−0.004	−0.010 to 0.000	0.110
Direct Effect 1	0.063	−0.007 to 0.131	0.069
Direct Effect 2	−0.086	−0.152 to −0.024	0.006
Direct Effect 3	0.085	0.022 to 0.147	0.008
Direct Effect 4	0.100	0.045 to 0.158	0.001
Total Indirect Effect	−0.003	−0.019 to 0.015	0.764
Total Direct Effect	0.163	0.048 to 0.277	0.005
Total Effect	0.160	0.050 to 0.281	0.006

Indirect Effect 1: Wife’s SP → Husband’s DEP → Husband’s CD; Indirect Effect 2: Husband’s SP → Husband’s DEP → Wife’s CD; Direct Effect 1: Wife’s SP → Husband’s CD; Direct Effect 2: Husband’s SP → Wife’s CD; Direct Effect 3: Wife’s SP → Wife’s CD; Direct Effect 4: Husband’s SP → Husband’s CD. SP, social participation; CD, chronic disease; DEP, depressive symptoms.

## 4 Discussion

To the best of our knowledge, this is the first study using nationally representative data to examine the association between spousal social participation and the number of chronic diseases in partners among Chinese adults aged ≥45 years, including gender differences. Our key findings were that: (1) spousal social participation was significantly associated with the number of chronic diseases in partners, with significant gender differences; and (2) the wife’s depressive symptoms significantly mediated this association. These findings underscore the importance of addressing how one spouse’s social participation relates to the other spouse’s health.

Previous studies have explored the relationship between spousal social participation and partner health outcomes. To the best of our knowledge, no existing research has specifically examined the association between a spouse’s social participation and the number of chronic diseases in their partner. Our study provides new insights by investigating this specific relationship, in line with prior research (31, 32). Based on a nationally representative sample, our study confirmed the association between a spouse’s social participation and the number of chronic diseases in the partner, after adjusting for potential confounding factors. A previous longitudinal study reported a significant association between spousal informal social participation and the partner’s mental health outcomes (33). In contrast to our findings, one study of older adult couples in China found that the husband’s social participation was significantly associated with the wife’s cognitive health, while the wife’s social participation showed no significant association with the husband’s cognitive health (34).

Our findings indicated gender differences in the association between a spouse’s social participation and chronic diseases in the partner, which is partially consistent with previous research. Earlier studies have shown a positive correlation between husbands’ social participation and wives’ cognitive function (35). However, the association between a wife’s social participation and the husbands’ health status has not yet been identified. This study is the first to identify the partner effect of spousal social participation with chronic diseases, highlighting gender differences within this relationship.

Finally, our study revealed that the wife’s depressive symptoms serve as significant mediators in the relationship between a spouse’s social participation and chronic diseases in the partner. Previous research has found that an individual’s social participation shows a significant association with their own depressive symptoms (36, 37). Additionally, spousal depressive symptoms are significantly associated with the other partner’s chronic diseases (38). These findings suggest that spousal social participation may relate to chronic disease in the other partner alongside individual depressive symptoms. Building on this foundation, our study is the first to systematically explore the complex relationships among spousal social participation, depressive symptoms, and chronic diseases.

The observed association between spousal social participation and a partner’s chronic disease can be explained through several mechanisms. First, according to emotional contagion theory, a close relationship between spouses facilitates the transfer of emotions and behaviors between partners (39). Individuals often integrate their partners’ health and well-being into their own perceptions, and the emotional and behavioral effects of one partner’s social participation may influence the other (40, 41). Second, the shared resource hypothesis posits that married couples typically share living environments and health resources, which can serve as either a foundation for promoting health or, when resources are scarce, as a source of health risks (42). When a spouse engages in social activities, it often leads to changes in health-related external resources, thereby affecting the partner’s health.

Notably, this association exhibited significant gender-related differences. A possible explanation lies in gender role theory, which emphasizes the distinct societal expectations for men and women within the family and society. These gender-based divisions of labor may contribute to disparities in how social participation influences spousal health (43). Higher levels of social participation among wives may disrupt traditional role divisions, leading to husbands taking on more household responsibilities, which could negatively affect their health (44). Conversely, higher levels of social participation among husbands may alleviate their wives’ caregiving responsibilities, thereby improving their quality of life and health outcomes. Moreover, studies have indicated that despite evolving gender role expectations in China, traditional gender role divisions persist, suggesting that gender role theory remains relevant in the Chinese societal context (45).

This study highlights the partner effect of social participation with chronic diseases among middle-aged and older adults in China, offering valuable insights for developing and optimizing public policies related to aging. First, the partner effect of social participation suggests that policymakers should design and implement policies promoting joint participation in health-related activities among couples. Encouraging mutual support between spouses for social participation can enhance health support within couples. Second, we advocate for strengthening social support networks for middle-aged and older adults. Given the significant impact of social participation on both individual and spousal chronic diseases, policymakers should focus on building and reinforcing support systems for middle-aged and older couples. This can be achieved by expanding community services, volunteer organizations, and senior activity centers to provide more opportunities for social participation. Third, the observed gender differences indicate that the effects of social participation on chronic diseases differ between men and women. This calls for gender-sensitive approaches in designing social participation and health promotion initiatives. For instance, policies targeting women’s social participation could include measures to alleviate household burdens, thereby reducing the potential negative effects of their participation on their husbands’ health.

This study has several limitations. First, we did not differentiate the relationships between specific types of social participation (e.g., volunteering, community engagement, social gatherings) and chronic diseases. Second, although we examined the association between social participation and the number of chronic diseases, disease severity—a critical indicator of clinical impact—remains unaddressed. Third, while our analysis of reverse causality (i.e., the potential influence of spousal chronic diseases on social participation) yielded no significant association, the observational design does not allow us to completely rule out such effects. Fourth, to maintain the simplicity of APIM, we did not adjust for several lifestyle habits (e.g., smoking, alcohol consumption, physical activity, and diet) and other potential confounders. This limitation may constrain the explanatory power of the analysis. Fifth, reliance on self-reported data for both chronic disease diagnoses and social participation introduces the risk of recall bias and misclassification. Finally, given that our findings are situated within China’s unique sociocultural context, the contextual generalizability of these results requires further validation in diverse settings. Future research should integrate richer datasets, employ advanced statistical techniques, and explore how various forms of social participation relate to chronic disease severity to enhance the precision and generalizability of the findings.

## 5 Conclusion

This study examined the relationship between a spouse’s social participation and chronic diseases in the partner using a nationally representative sample from China. The results indicated that spousal social participation is associated with the partner’s chronic diseases among Chinese middle-aged and older adults, and that this association is gender-dependent. Therefore, policymakers should consider the impact of social participation on both individual and spousal health when designing health promotion policies for middle-aged and older adults. Special attention should be given to gender differences in the development of gender-sensitive health interventions aimed at encouraging couples to jointly engage in health management.

## Data Availability

Publicly available datasets were analyzed in this study. This data can be found at: https://charls.pku.edu.cn/en/.
