# Supplementary material for: Spousal associations between social participation and chronic diseases among Chinese middle-aged and older adults: an actor-partner interdependence model analysis
Source: Front Public Health. 2025 Aug 8;13:1576933. doi: 10.3389/fpubh.2025.1576933 (PMC12370478; doi:10.3389/fpubh.2025.1576933)
Supplement: Supplementary file 1 [file Supplementary_file_1.docx]

**Supplementary Materials**

**Table S1.** Statistical information of key variables from 2013, 2015 and 2018.

| Variables | Wife (n=3072)  N (%) or Mean (SD) | Husband (n=3072)  N (%) or Mean (SD) |
| --- | --- | --- |
| Social Participation (2013) | 0.91 (1.06) | 1.04 (1.15) |
| Social Participation (2015) | 0.90 (1.13) | 1.04 (1.25) |
| Social Participation (2018) | 0.85 (1.12) | 0.92 (1.15) |
| Chronic Disease (2013) | 1.36 (1.37) | 1.25 (1.32) |
| Chronic Disease (2015) | 1.54 (1.49) | 1.40 (1.43) |
| Chronic Disease (2018) | 2.43 (1.92) | 2.25 (1.88) |
| Depressive Symptoms (2013) | 8.44 (5.96) | 6.51 (5.00) |
| Depressive Symptoms (2015) | 8.78 (6.57) | 6.57 (5.76) |
| Depressive Symptoms (2018) | 9.49 (6.73) | 7.22 (5.87) |

**Note**: Continuous and categorical variables were expressed as mean (standard deviation) and number (proportion), respectively.

| **Table S2.** Actor and partner effect coefficients of the actor-partner interdependence model. | | | | |
| --- | --- | --- | --- | --- |
| Pathways Estimate | β | SE | 95% CI | P Value |
| Partner Effect Coefficient |  |  |  |  |
| Wife’s SP_2013_ → Husband’s CD_2018_ | 0.065 | 0.033 | 0.001 to 0.129 | 0.048 |
| Husband’s SP_2013_ → Wife’s CD_2018_ | -0.092 | 0.031 | -0.152 to -0.032 | 0.003 |
| Actor Effect Coefficient |  |  |  |  |
| Wife’s SP_2013_ → Wife’s CD_2018_ | 0.086 | 0.033 | 0.021 to 0.151 | 0.009 |
| Husband’s SP_2013_ → Husband’s CD_2018_ | 0.086 | 0.030 | 0.026 to 0.145 | 0.005 |

**Notes**: The parameters in Table S2 correspond to Figure 3; The subscripts refer to the assessment year point; β= Standardized Coefficient; SE= Standard Error; CI = confidence interval; Model goodness-of-fit indices: X^2^ = 554.356; df = 26; p value = 0.000; comparative fit index (CFI) = 0.677; Tucker-Lewis index (TLI) = 0.478; root mean square error of approximation (RMSEA) = 0.081; The model was adjusted for baseline individual-level age, activities of daily living (ADL), work status, region, and household assets.

| **Table S3.** Actor and partner effect coefficients in the longitudinal mediation model with wives' depressive symptoms as a mediator. | | | | |
| --- | --- | --- | --- | --- |
| Pathways Estimate | β | SE | 95% CI | P Value |
| Mediating Pathways |  |  |  |  |
| Wife’s SP_2013_ → Wife’s DEP_2015_ | -0.319 | 0.112 | -0.538 to -0.107 | 0.004 |
| Wife’s DEP_2015_ → Husband’s CD_2018_ | 0.029 | 0.005 | 0.019 to 0.039 | 0.000 |
| Husband’s SP_2013_ → Wife’s DEP_2015_ | -0.201 | 0.101 | -0.408 to -0.010 | 0.046 |
| Wife’s DEP_2015_ → Wife’s CD_2018_ | 0.073 | 0.005 | 0.063 to 0.083 | 0.000 |
| Partner Effect Coefficient |  |  |  |  |
| Wife’s SP_2013_ → Husband’s CD_2018_ | 0.075 | 0.036 | 0.013 to 0.152 | 0.037 |
| Husband’s SP_2013_ → Wife’s CD_2018_ | -0.077 | 0.030 | -0.134 to -0.018 | 0.009 |
| Actor Effect Coefficient |  |  |  |  |
| Wife’s SP_2013_ → Wife’s CD_2018_ | 0.110 | 0.032 | 0.048 to 0.170 | 0.001 |
| Husband’s SP_2013_ → Husband’s CD_2018_ | 0.091 | 0.032 | 0.028 to 0.152 | 0.004 |

**Notes**: The parameters in Table S3 correspond to Figure 4; The subscripts refer to the assessment year point; β= Standardized Coefficient; SE= Standard Error; CI = confidence interval; Model goodness-of-fit indices: X^2^ = 565.943; df = 30; p value = 0.000; comparative fit index (CFI) = 0.756; Tucker-Lewis index (TLI) = 0.553; root mean square error of approximation (RMSEA) = 0.076; The model was adjusted for baseline individual-level age, activities of daily living (ADL), work status, region, and household assets.

| **Table S4.** Actor and partner effect coefficients in the longitudinal mediation model with husbands' depressive symptoms as a mediator. | | | | |
| --- | --- | --- | --- | --- |
| Pathways Estimate | β | SE | 95% CI | P Value |
| Mediating Pathways |  |  |  |  |
| Wife’s SP_2013_ → Husband’s DEP_2015_ | 0.017 | 0.099 | -0.173 to 0.222 | 0.861 |
| Husband’s DEP_2015_ → Husband’s CD_2018_ | 0.088 | 0.006 | 0.075 to 0.100 | 0.000 |
| Husband’s SP_2013_ → Husband’s DEP_2015_ | -0.161 | 0.090 | -0.340 to 0.013 | 0.074 |
| Husband’s DEP_2015_ → Wife’s CD_2018_ | 0.025 | 0.006 | 0.012 to 0.038 | 0.000 |
| Partner Effect Coefficient |  |  |  |  |
| Wife’s SP_2013_ → Husband’s CD_2018_ | 0.063 | 0.035 | -0.007 to 0.131 | 0.069 |
| Husband’s SP_2013_ → Wife’s CD_2018_ | -0.086 | 0.031 | -0.152 to -0.024 | 0.006 |
| Actor Effect Coefficient |  |  |  |  |
| Wife’s SP_2013_ → Wife’s CD_2018_ | 0.085 | 0.032 | 0.022 to 0.147 | 0.008 |
| Husband’s SP_2013_ → Husband’s CD_2018_ | 0.100 | 0.029 | 0.045 to 0.158 | 0.001 |

**Notes**: The parameters in Table S4 correspond to Figure 5; The subscripts refer to the assessment year point; β= Standardized Coefficient; SE= Standard Error; CI = confidence interval; Model goodness-of-fit indices: X^2^ = 582.778; df = 30; p value = 0.000; comparative fit index (CFI) = 0.740; Tucker-Lewis index (TLI) = 0.523; root mean square error of approximation (RMSEA) = 0.077; The model was adjusted for baseline individual-level age, activities of daily living (ADL), work status, region, and household assets.
